# Integrative analysis of differential miRNA and functional study of miR-21 by seed-targeting inhibition in multiple myeloma cells in response to berberine

**DOI:** 10.1186/1752-0509-8-82

**Published:** 2014-07-07

**Authors:** Xiaochuang Luo, Jingyi Gu, Rongxuan Zhu, Maoxiao Feng, Xuejiao Zhu, Yumin Li, Jia Fei

**Affiliations:** 1 Department of Biochemistry and Molecular Biology, Medical College of Jinan University, Guangzhou 510632, China; 2 Department of Clinical Medicine, Medical College of Jinan University, Guangzhou 510632, China

**Keywords:** Berberine, miRNA-21, Multiple myeloma, Programmed cell death 4, Bioinformatic, Tumor protein p53, Cell cycle, Apoptosis

## Abstract

**Background:**

Berberine is a natural alkaloid derived from a traditional Chinese herbal medicine. It is known to modulate microRNA (miRNA) levels, although the mechanism for this action is unknown. Here, we previously demonstrate that the expression of 87 miRNAs is differentially affected by berberine in multiple myeloma cells. Among 49 miRNAs that are down-regulated, nine act as oncomirs, including miR-21. Integrative analysis showed that 28 of the down-regulated miRNAs participate in tumor protein p53 (TP53) signaling and other cancer pathways. miR-21 is involved in all these pathways, and is one of the most important oncomirs to be affected by berberine in multiple myeloma cells.

**Results:**

We confirmed that berberine down-regulated miRNA-21 expression and significantly up-regulated the expression of programmed cell death 4 (*PDCD4*), a predicted miR-21 target. Luciferase reporter assays confirmed that *PDCD4* was directly regulated by miR-21. Bioinformatic analysis revealed that the miR-21 promoter can be targeted by signal transducer and activator of transcription 3 (STAT3). Down-regulation of interleukin 6 (IL6) by berberine might lead to inhibition of miR-21 transcription through STAT3 down-regulation in multiple myeloma. Furthermore, both berberine and seed-targeting anti-miR-21 oligonucleotide induced apoptosis, G2-phase cell cycle arrest and colony inhibition in multiple myeloma cell lines. Depletion of PDCD4 by short interfering RNA could rescue berberine-induced cytotoxicity in multiple myeloma cells.

**Conclusions:**

Our results suggest that berberine suppresses multiple myeloma cell growth, at least in part, by down-regulating miR-21 levels possibly through IL6/STAT3. This led to increased *PDCD4* expression, which is likely to result in suppression of the p53 signaling pathway. These findings may also provide new mechanistic insight into the anti-cancer effects of certain compounds in traditional Chinese herbal medicines.

## Background

Multiple myeloma (MM) is a clonal B cell malignancy characterized by proliferation of plasma cells (PCs) within the bone marrow (BM). Globally, its incidence varies from 1 per 100,000 people in China to about 4 per 100,000 people in most developed countries [1,2]. MM is characterized by profound genomic instability involving both numerical and structural chromosomal aberrations of potential prognostic relevance [1,2]. Nearly half of MM tumors are hyperdiploid (HD) with multiple trisomies of non-random odd-numbered chromosomes, a low prevalence of chromosomal translocations involving the immunoglobulin heavy chain (IgH) locus at 14q32 and chromosome 13 deletion [3]. It has been suggested that chromosomal abnormalities and other types of genetic or epigenetic alterations might contribute to miRNA deregulation in cancer [4-6].

miRNAs are a newly discovered class of endogenous non-coding small RNAs that regulate gene expression through degrading target mRNAs and/or suppressing their translation by binding to the 3′-untranslated region (3′-UTR) of target genes. Bioinformatic predictions indicate that 30% of all human genes are regulated by miRNAs. Thus, miRNAs are involved in a variety of biological processes, from development and differentiation to survival, apoptosis, and senescence [7-9].

Accumulating evidence suggests that miRNAs that are significantly over-expressed in tumors may be a novel class of oncogene. Termed “oncomirs”, these oncogene miRNAs usually promote tumor development by negatively regulating tumor suppressor genes that control various biological processes. Therefore, altering oncomir expression might be a valuable strategy for cancer treatment [10,11].

Differential miRNA expression and high levels of oncomirs, including miR-21, miR-155, miR-17-92, and miR-125b, have been reported in MM. miR-21 is frequently over-expressed in MM and is involved in proliferation, apoptosis, cell cycle, drug-resistance, and pathogenesis [6,12-15]. Loffler et al. demonstrated that interleukin-6 (IL6) regulates miR-21 transcription in IL6-dependent human myeloma cell lines (HMCLs) through a signal transducer and activator of transcription 3 (STAT3)-related mechanism. Importantly, ectopic expression of miR-21 was sufficient to sustain growth of IL6-dependent MM cells in the absence of IL6 [16]. This evidence indicates that miR-21 is an important oncomir in MM.

Berberine (BB), an alkaloid that was initially isolated from Chinese herbs, is currently used as a traditional medicine to treat diarrhea caused by bacteria, although the mechanism for this action is unknown. Accumulating evidence suggests that BB also elicits anti-cancer effects by inhibiting cell growth and inducing apoptosis in a variety of cancer cell lines [17-20]. Animal studies have shown that BB can inhibit chemical-induced carcinogenesis, tumor promotion, and tumor invasion [21,22]. Recent studies also show that BB exerts anti-cancer effects by inhibiting proliferation and reproduction of certain tumorigenic microorganisms and viruses, such as *Helicobacter pylori* and hepatitis B. BB can also regulate the transcription of some oncogenes and carcinogenesis-related genes via interactions with DNA and RNA. Furthermore, BB is a broad-spectrum enzyme inhibitor that affects *N*-acetyltransferase, cyclooxygenase-2, and topoisomerase activities, as well as gene expression and protein synthesis [23]. Thus, BB can regulate many oncogenic mRNAs and proteins. However, whether BB can regulate miRNAs remains unknown.

miRNAs in animals have a highly conserved 5′-end sequence consisting of 7–8 nt called the seed sequence. The seed sequence binds with 100% complementarity to the target mRNA and is a key feature in the recognition between a miRNA and its target mRNA [24]. Inhibition of the seed sequence leads to a loss of mature miRNA function, and is the target of anti-miRNA oligonucleotides (AMOs) [25,26].

In this research, we performed microarray analysis to explore the possibility that BB regulates miRNA expression. Our results show that BB differentially regulates the expression of a number of miRNAs. Forty-nine miRNAs were down-regulated, of which 28 were shown by KEGG analysis to be involved in p53 signaling, the cell cycle and other cancer pathways. Of the 49 miRNAs, miR-21 had the most target genes and participates in all the signaling pathways and can, therefore, be considered as one of the most important oncomirs. The role of miR-21 in MM was further investigated with the use of AMO-miR-21. Our findings provide new insight into anti-cancer mechanisms of traditional Chinese herbal medicines and provide evidence that they are effective in treating cancer.

## Methods

### Microarray analysis of miRNA expression

Based on our preliminary study, the MM cell line, RPMI-8266, was treated with 75 μM BB for 48 h. Total miRNA from 1 × 10^8^ cells was isolated and labeled using an mirVANA™ miRNA Isolation kit and mirVANA™ miRNA labeling kit (Ambion, Austin, TX, USA).. Samples (4 μg) labeled with Cy3/Cy5 were hybridized on miRNA microarrays (CSC-GE-3, chipscreen biosciences, Shenzhen, China). After air drying, each chip was scanned with a Generation III array scanner (Amersham Pharmacia). Data analyses were performed using Imagequant 5.0 (Array Vision 6.0).

### Bioinformatic analysis

miRFocus software (http://mirfocus.org), developed by LC Science USA, was used to analyze miRNA-target gene pathways and to determine related miRNA annotations (Additional file 1: Figure S1).

### Oligonucleotides

An anti-miR-21 oligonucleotide (AMO-miR-21) was designed according to sequence complementary to mature miRNA-21: AMO-miR-21, 5′-ATAAGCTA-3′ (8 bp). A control scramble AMO (SCR) 5′ -TCATACTA-3′ (8 bp) was also synthesized (Additional file 1: Figure S2). All oligodeoxynucleotides were chemically synthesized and modified with phosphorothioate and/or fluorescein isothiocyanate (FITC) by the Shanghai Sangon Bio-engineering Company, China. The siRNA sequence of *PDCD4* (siPDCD4) was 5′-AAGGUGGCUGGAACAUCUAUU-3′. The RNA duplexes were synthesized and purified by Shanghai GenePharma Company, China.

### Cell lines, transfection and cell culture reagents

MM cell lines (RPMI-8266 and U226) were obtained from the Shanghai Institute of Cell Biology, China. The cells were cultured in RPMI containing 25 mM HEPES, 10% fetal bovine serum (FBS), 0.05 mM 2-mercaptoethanol, 1 mM sodium pyruvate, 2 mM L-glutamine, 100 U/mL penicillin, and 50 U/mL streptomycin. The cells were grown in RPMI-1640 medium containing 10% fetal calf serum (FCS) at 37°C in a 5% CO_2_ humidified atmosphere (Thermo FORMA 3110).

BB was purchased from Sigma-Aldrich. RPMI-8266 and U226 cells in the exponential phase of growth were seeded in 96- or 24-well plates (Costar) and transfected with 0.5 μM AMO-miR-21 using Lipofectamine 2000 (Invitrogen) in serum-free RPMI-1640. *PDCD4* siRNA and control SCR (100 nM) were transfected into RPMI-8266 and U226 cells using Lipofectamine 2000 according to the manufacturer’s instructions.

### Luciferase reporter assays

The full-length human *PDCD4* 3′-UTR (1917 bp) was PCR-amplified from cDNA with the following primers: 5′-ccg*ctcgag*ATATAAGAACTCTTGCAGTCT-3′ and 5′-ataagaat*gcggccgc*ACAGAGGATCTTTACATGTTTA-3′ containing NotI and XhoI restriction site overhangs, respectively (indicated in italic type). The amplified product, which contains one putative miR-21 binding site, was cloned into psiCHECK-2 (Promega) immediately downstream of the Renilla luciferase reporter gene and was named PDCD4 3′-UTR. Site-directed mutagenesis was performed using the QuikChange II XL site-directed mutagenesis kit (Stratagene) to change three nucleotides in the seed sequence, in which ATAAGCTA was substituted by TAGCTACT. The mutant plasmid was named PDCD4-mut- 3′-UTR.

RPMI-8266 cells were cotransfected with 100 nM miR-21 or 0.5 μM AMO-miR-21 together with PDCD4 3′-UTR or PDCD4*-*mut-UTR and assayed for luciferase activity 24 h post-transfection using the Dual-Luciferase Reporter Assay System (E1910, Promega). For each sample, firefly luciferase activity was normalized against Renilla luciferase activity.

### Real-time PCR assay

RPMI-8266 and U226 cells were treated with 75 μM and 120 μM BB, respectively. Total RNA was extracted in TRIzol (Invitrogen). The levels of miR-21 and U6 small nuclear RNA (snRNA) were determined using a miRNA RT-PCR Quantitation Kit (Shang Hai Gene Pharma Company). U6 snRNA was used as the internal control, and the fold-change in miR-21 expression was calculated using the 2^−ΔΔCT^ method.

RPMI-8266 cells were transfected with 0.5 μM AMO-miR-21 using Lipofectamine 2000 and cultured for 48 h. Levels of *PDCD4* mRNA were determined using SYBR-Green real-time PCR assays. *PDCD4* primers used were 5′-CCAAAGAAAGGTGGTGCA-3′ and 5′-TGAGGTACTTCCAGTTCC-3′ and GAPDH primers were 5′-CAACGGATTTGGTCGTATT-3′ and 5′-CACAGTCTTCTGG GTGGC-3′. *PDCD4* mRNA levels were normalized to those of *GAPDH*.

### Western blot analysis and enzyme-linked immunosorbent assay (ELISA)

Cells were lysed in radioimmunoprecipitation assay (RIPA) buffer in the presence of proteinase inhibitor (Biocolor BioScience & Technology Company, Shanghai, China). Cell lysates (30 μg) were denatured in Laemmli sample buffer (Bio-Rad) for 5 min at 95.1°C, separated by 10% SDS-PAGE and transferred to nitrocellulose membranes. Membranes were blocked with 5% (w/v) fat-free milk in phosphate-buffered saline (PBS) and 0.5% (v/v) Tween-20 for 1 h, and then incubated with anti-PDCD4 antibody (Cell Signaling Technology) at room temperature for 2 h. After washing, membranes were incubated with horseradish peroxidase-conjugated secondary antibody. Signals were visualized with enhanced chemiluminescence (ECL) (BeyoECL Plus, Beyotime), and analyzed using a BI-2000 system (Beyotime, Haimen, Jiangsu province, China),.

RPMI-8266 and U226 cells were treated with 75 μM and 120 μM BB, respectively.

IL6 protein levels in supernatants were determined using a human IL6 ELISA kit (R&D Systems).

### MTT assay

RPMI-8266 cell viability was determined by 3-(4,5-dimethylthiazol-2-yl)-2,4- diphenyl-tetrazolium bromide (MTT) assays. Briefly, cells were seeded at a density of 1 × 10^5^ cells/ml in 96-well plates (100 μl/well). The cells were treated with BB (75 μM) or AMO-miR-21 (0.5 μM). At 48 h post-treatment, 20 μl MTT stock solution (5 mg/ml) was added to each well, and the plate was incubated for 4 h at 37°C. The media was then removed, and dimethyl sulfoxide (DMSO) (150 μl) was added to dissolve the blue formazan crystals produced by viable cells. Cell viability was assessed by measuring the absorbance at 570 nm on a Bio-Rad microtiter plate reader.

### Colony assay

The colony assay for dispersed single cells was performed to measure the capacity of cells to form colonies. Cells treated with BB (75 μM) or AMO-miR-21 (0.5 μM) were seeded onto a 24-well plate (2 × 10^3^ cells per well) and mixed thoroughly with 0.9% methylcellulose solution in RPMI-1640 containing 20% FBS. Single cells were randomly and evenly distributed throughout each well. Colonies were formed during incubation for 1–2 weeks at 37°C in a 5% CO_2_ humidified atmosphere. Light microscopy was used to observe and count colonies containing more than 50 cells.

### Flow cytometry

Flow cytometry (Coulter Elite; Fullerton, CA, USA) was performed to analyze cell cycle profiles and levels of apoptosis. For cell cycle analysis, cells were collected, rinsed twice with PBS, fixed in 70% ethanol for 1 h at 4°C and stained with propidium iodide (PI) solution (50 μg/ml) containing RNAse A (200 ug/ml). Cell cycle was analyzed using flow cytometry according to DNA content. To analyze apoptosis, cells were stained with fluorescein isothiocyanate (FITC)-conjugated annexin V and PI. For each sample, data from approximately 10,000 cells were recorded in the list mode on logarithmic scales. Apoptotic and necrotic cells were analyzed by performing quadrant statistics on PI-negative/annexin V-positive cells and PI/annexin V double-positive cells, respectively.

## Results

### BB modulates miRNA expression in MM cells

**Figure 1:**
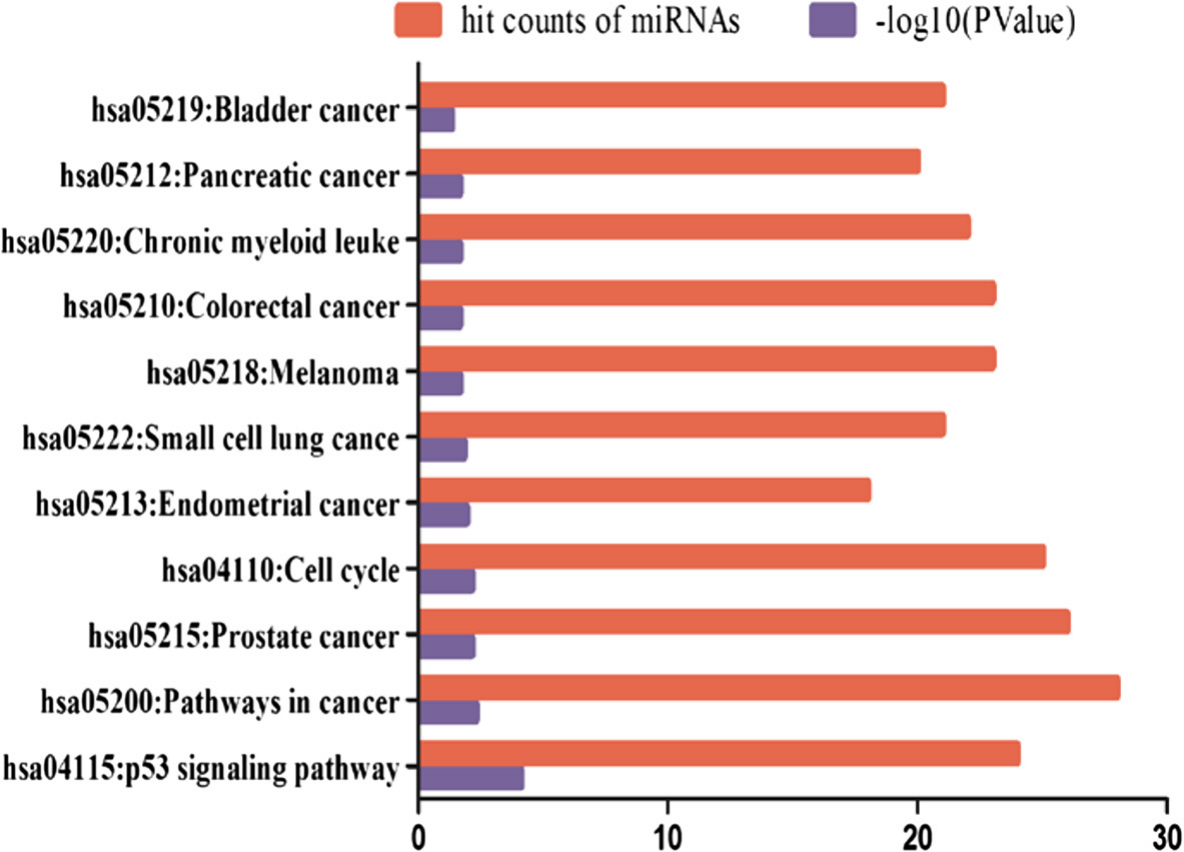
**Ingenuity analysis of miRNA predictive pathways.** Among the 49 down-regulated miRNAs, 28 were shown by mirFocus to be involved in p53 signaling, cell cycle and other cancer pathways. miR-21 participates in all the above signaling pathways, and can be considered as one of the most important oncomirs in the effects of BB on MM.

Of the 1152 miRNAs represented on the microarray, 87 were differentially expressed between BB-treated and control cells (Additional file 1: Figure S3); 49 were down-regulated, and 38 were up-regulated compared to control. Further analysis revealed that nine of the 49 down-regulated miRNAs were potential oncomirs, and three of the 38 up-regulated miRNAs are considered tumor suppressor genes (Additional file 2: Table S1 and Additional file 2: Table S2). Among the 49 down-regulated miRNAs, mirFocus software identified 28 that are involved in p53 signaling, the cell cycle and other cancer pathways (Figure 1). Of these, miR-21, has the most identified target genes and participates in all the above signaling pathways. Thus mir-21 can be considered as one of the most important oncomirs. These results suggest that BB suppression of MM might involve miRNA-mediated gene expression.

### Regulation of miRNA-21, IL6 and PDCD4 levels by berberine and the effects of exogenous IL6 on miRNA-21 expression in MM cells

**Figure 2:**
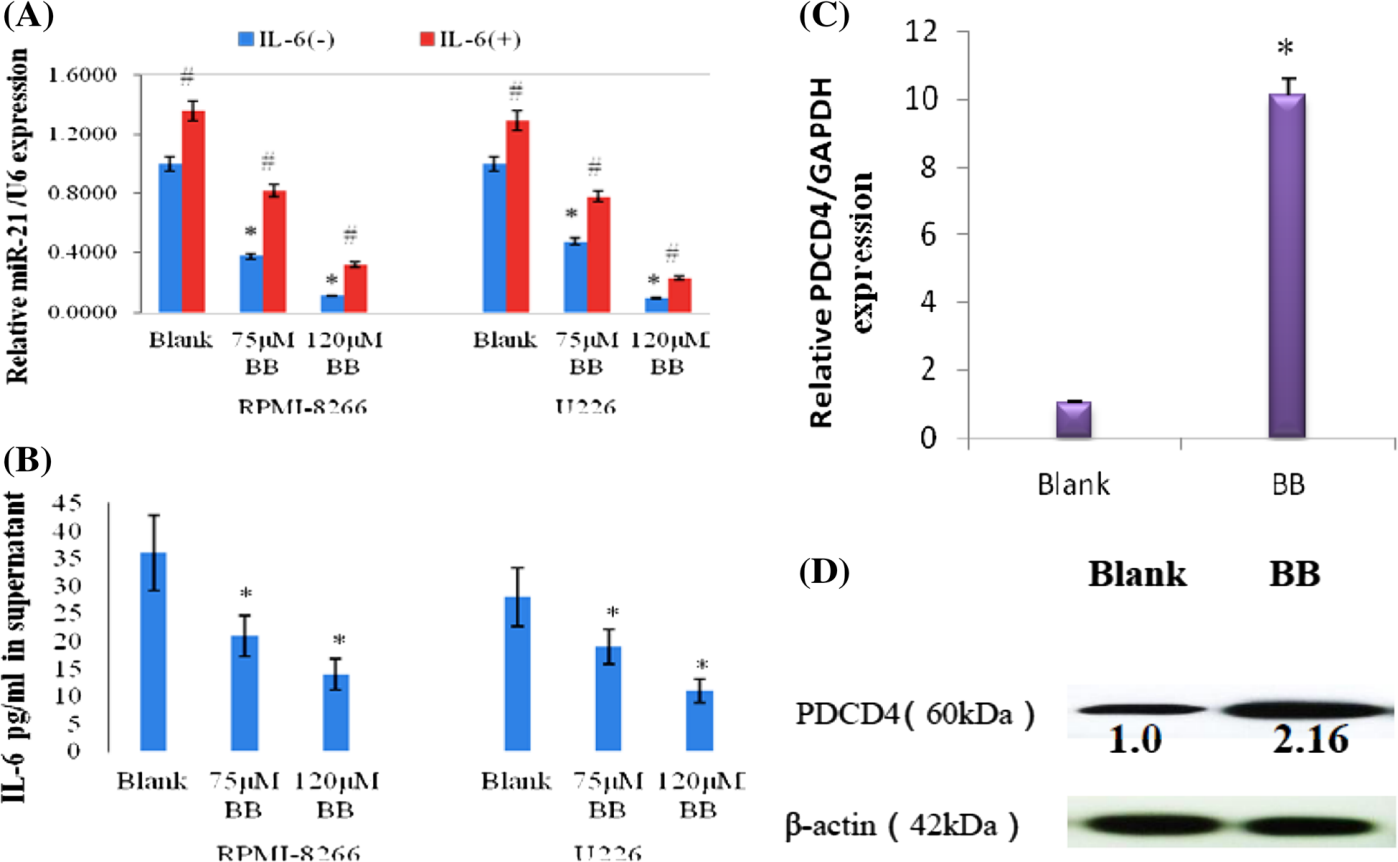
**Effect of BB on levels of miRNA-21, IL6, and PDCD4 and the effect of exogenous IL6 on BB-induced miRNA-21 expression.** RPMI-8266 and U226 cells were treated with 75 μM and 120 μM BB, respectively, in the absence or presence of IL6 (0.5 ng/ml) and harvested 48 h after treatment. Total RNA and protein were isolated and analyzed for levels of miRNA-21 and PDCD4. **(A)** miR-21 and U6 snRNA levels were determined by quantitative real-time PCR in the absence or presence of IL6. **(B)** The IL6 protein levels in supernatants were determined using an ELISA kit. **(C)** Relative PDCD4 mRNA levels were measured with SYBR-Green real-time PCR. **(D)** PDCD4 protein levels were assessed by western blot analysis. *p < 0.01 vs. control. The data show that BB significantly down-regulated miR-21 and IL6 levels in RPMI-8266 and U226 cells and up-regulated PDCD4 mRNA and protein levels in 8266 cells. Notably, exogenous IL6 could attenuate the reduction in miRNA-21 expression induced by BB.

To validate down-regulation of miR-21 by BB and to investigate the effect of exogenous IL6 on BB-modulated miRNA-21 expression in MM, RPMI-8266 and U226 cells were treated without or with 75 μM or 120 μM BB, respectively, in the absence or presence of IL6 (0.5 ng/ml). Total RNA and/or protein was isolated and analyzed for the expression of miR-21 and PDCD4 using real-time PCR and western blot analyses. As shown in Figure 1, BB treatment significantly down-regulated miR-21 levels in a dose-dependent manner, and exogenous IL6 somewhat rescued BB-mediated down-regulation of miR-21 (Figure 2A). Interestingly, BB also reduced the level of IL6 in supernatant (Figure 2B). IL6 is an important oncogene in MM and is involved in miR-21 transcription [16]. These results confirmed that BB down-regulates miR-21 and IL6 and consequently the miRNA target gene, *PDCD4* was up-regulated (Figure 2C, 2D). Meanwhile, exogenous IL6 could rescue BB-induced miRNA-21 down-regulation in MM cells.

### BB down-regulates STAT3 mRNA levels in MM cells

Bioinformatic analysis showed that sequence upstream of the miR-21 gene contained two putative STAT3 binding sites (Additional file 1: Figure S4A). To investigate whether BB affects STAT3 mRNA levels, RPMI-8266 and U226 cells were treated with 75 μM and 120 μM BB, respectively. STAT3 mRNA levels were significantly decreased (Additional file 1: Figure S4B). STAT3 is recruited to the miR-21 regulatory region in response to IL6 [16]; therefore, BB-mediated down-regulation of IL6 might cause transcriptional inhibition of miR-21 via reduced STAT3 action.

### *PDCD4* is a direct target of miRNA-21

Because BB treatment reduced miRNA-21 levels in MM cells, we next examined whether BB also regulated the expression of genes targeted by miRNA-21. We first focused on the *PDCD4* gene, which is known to be targeted by miR-21. AMO-miR-21 was transfected into RPMI-8266 cells to knock down endogenous mir-21. As shown in Additional file 1: Figure S5A and S5B, *PDCD4* mRNA and protein levels were significantly increased in AMO-miR-21-transfected cells compared with control-transfected cells, suggesting that *PDCD4* is regulated by miR-21.

We performed luciferase reporter assays to determine whether *PDCD4* is a direct target of miR-21. The luciferase reporter plasmid containing the 3′-UTR of *PDCD4* (PDCD4-3′UTR) or a mutant PDCD4 3′-UTR containing a mutation in the putative miR-21 binding site (PDCD4-mut-3′UTR) were co-transfected with AMO-miR-21 or a miRNA-21 mimic. The firefly luciferase plasmid was used as an internal control. As shown in Additional file 1: Figure S5C, over-expression of the miR-21 mimic significantly suppressed luciferase activity, whereas transfection of AMO-miR-21 significantly increased luciferase activity in cells transfected with PDCD4-3′UTR. In contrast, luciferase activities in cells transfected with PDCD4-mut-3′UTR were not significantly changed by either over-expression or knockdown of miR-21 (Additional file 1: Figure S5C,S5D). These results indicate that *PDCD4* might be a direct target of miR-21.

### Transfection efficiency and localization of AMO-mir-21 in RPMI-8266

AMO-mir-21 was modified with FITC, and transfected into RPMI-8266 cells using Lipofectamine 2000. High levels of AMO-mir-21-FITC were detected, mainly in the cytoplasm (Additional file 1: Figure S6A). Flow cytometry showed that at 24 h and 48 h post-transfection 95.27% and 85.8% of cells, respectively, were FITC-positive (Additional file 1: Figure S6B).

### BB and AMO-miR-21 inhibit cell growth and induce apoptosis

**Figure 3:**
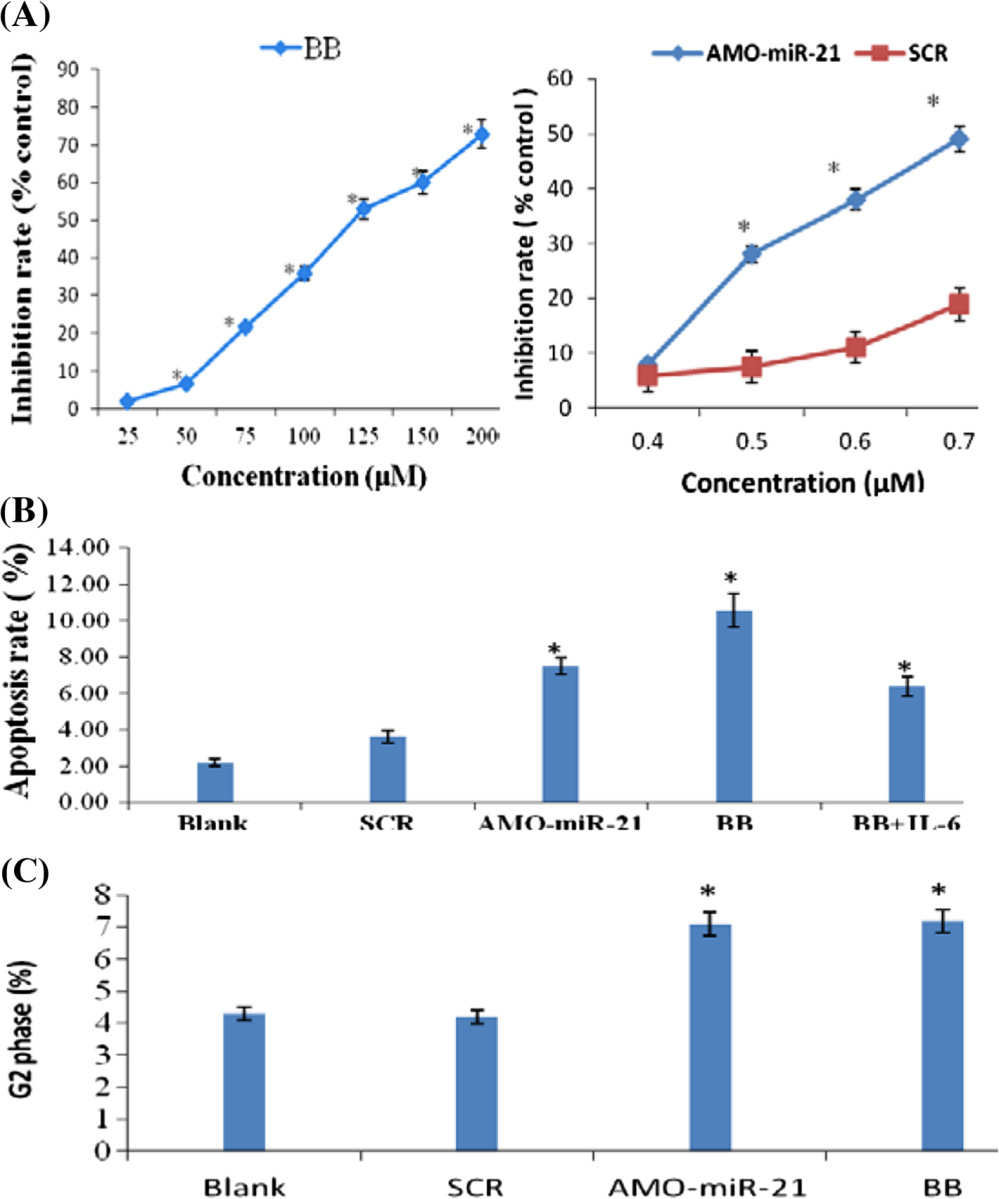
**BB and AMO-miR-21 inhibit cell viability and induce apoptosis and G2 phase cell cycle arrest.** RPMI-8266 cells were treated with various concentrations of BB and AMO-miR-21 then plated in 96-well plates in medium containing 10% FCS and cultured for another 48 h. Cell viability was assessed by triplicate MTT assays. **(A)** BB and AMO-miR-21. *p < 0.01 vs. blank and Scramble controls. Both BB and AMO-miR-21 inhibited cell viability in a dose-dependent manner. RPMI-8266 cells were treated with 75 μM BB or 0.5 μM AMO-miR-21. Cells were then stained with FITC-conjugated annexin V and PI for 15 min., and then analyzed by flow cytometry. **(B)** Both BB and AMO-miR-21 promoted apoptosis and IL6 could reduce BB-induced apoptosis. *p < 0.01 vs. control. **(C)** Both BB and AMO-miR-21 significantly induced G2-phase cell cycle arrest. *p < 0.01 vs. blank or SCR controls.

To determine the effects of BB on MM cell growth via miR-21, RPMI-8266 cells were treated with BB (75 μM) or AMO-miR-21 (0.5 μM) as described in Materials and Methods. Cell viability was assessed by triplicate MTT assays. As shown in Figure 3A, treatment with BB at 50 μM or higher significantly inhibited cell proliferation. Similar results were also observed when miR-21 was knocked down by AMO-miR-21 (Figure 3A), and these effects were dose-dependent. Thus, both BB and AMO-miR-21 inhibited MM cell viability, indicating that the BB-mediated cell death might involve miR-21 down-regulation.

We next investigated whether BB treatment or miR-21 inhibition could induce apoptosis in MM cells. To this end, RPMI-8266 cells were treated with BB or transfected with AMO-miR-21. The cells were then stained with annexin V and PI and analyzed by flow cytometry. As shown in Figure 3B and Additional file 1: Figure S7, both treatments significantly increased the number of apoptotic cells, indicating that BB treatment and miR-21 inhibition induce apoptosis.

### BB and AMO-miR-21 induce G2-phase cell cycle arrest

To investigate the effect of BB treatment or miR-21 inhibition on the cell cycle, RPMI-8266 cells were treated with BB or transfected with AMO-miR-21 and then subjected to cell cycle analysis by flow cytometry. As shown in Figure 3C and Additional file 1: Figure S8, BB treatment resulted in an accumulation of cells in the G2/M phase. Interestingly, miR-21 inhibition by AMO-miR-21 produced almost identical effects on the cell cycle to those observed following BB treatment. These results indicate that BB-induced G2/M arrest in MM cells is at least partially mediated via miR-21 down-regulation.

### BB and AMO-miR-21 suppress colony formation

**Figure 4:**
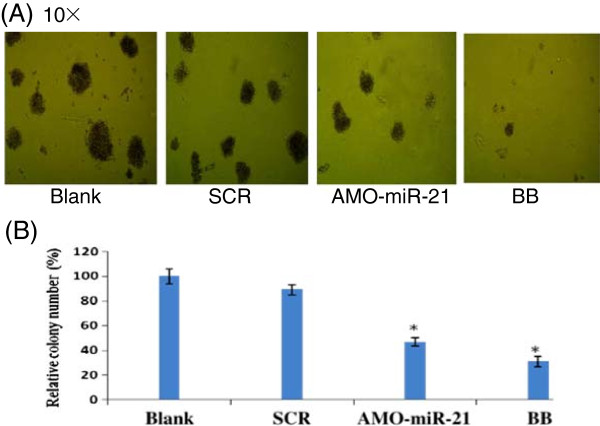
**BB and AMO-miR-21 suppress colony formation.** Colony growth capacity was assessed by methylcellulose colony formation assays. **(A)** Images of representative colony formation. **(B)** After incubation for 1 week, RPMI-8266 cells treated with 75 μM BB or 0.5 μM AMO-miR-21 were observed under a light microscope and the number of colonies counted. Three independent experiments were carried out.

Colony growth is closely related to neoplastic capacity. To investigate the effect of BB and AMO-miR-21 on colony formation in RPMI-8266 cells, colony growth assays were performed. One week post-treatment, we found that RPMI-8266 cells treated with AMO-miR-21 or BB showed fewer colonies, compared to the control groups (Figure 4). These results indicate that both BB and miR-21 knockdown had an inhibitory effect on the malignant growth capacity of MM cells.

### Testing siRNAs for their ability to silence *PDCD4*

**Figure 5:**
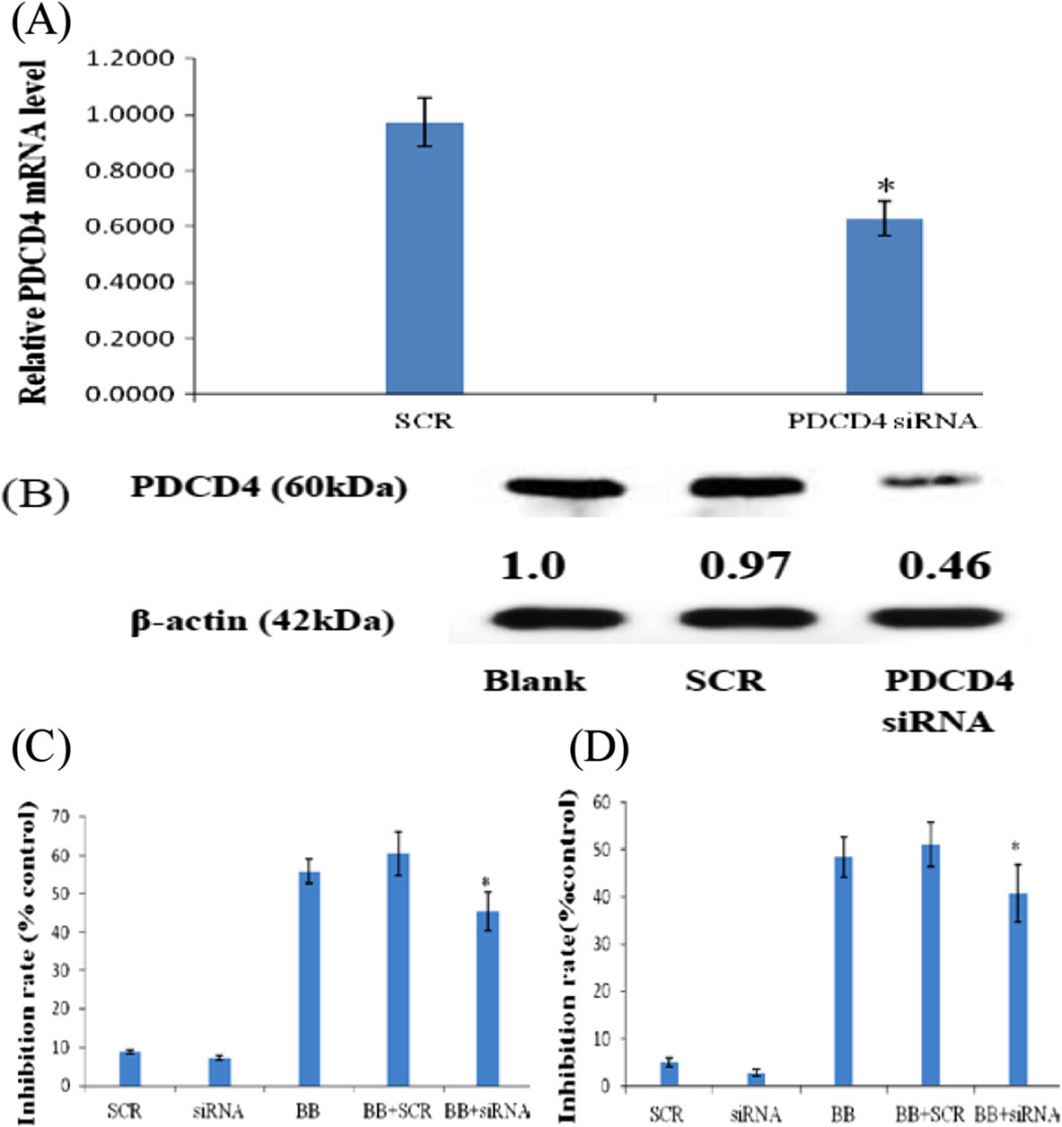
**siRNA down-regulation of PDCD4 expression and rescue of BB-induced growth inhibition.** RPMI-8266 cells were treated with 100 nM PDCD4 siRNA for 6 h, and harvested 48 h after treatment. Total RNA and protein were isolated and analyzed for PDCD4 expression levels. **(A)** PDCD4 mRNA levels were determined by SYBR-Green real-time PCR. **(B)** PDCD4 protein levels were determined by western blot analysis. PDCD4 siRNA significantly down-regulated PDCD4 levels in RPMI-8266 cells. *p < 0.01, vs. control. RPMI-8266 and U226 cells were pre-treated as above and then plated in 96-well plates in medium containing 10% FCS for a further 72 h in the presence of BB (75 μM). Cell viability was assessed by triplicate MTT assays. The results indicate that PDCD4 siRNA can rescue BB-induced growth inhibition. **(C)** RPMI-8266 cells. **(D)** U226 cells. *p < 0.01 vs. control.

To examine whether miR-21 down-regulation is associated with BB-induced cytotoxicity, we further studied the effect of *PDCD4* knockdown in MM cells. We designed three siRNAs against the *PDCD4* gene and evaluated their knockdown efficiency using real-time PCR analysis. We found that one siRNA efficiently knocked down *PDCD4* mRNA and down-regulated PDCD4 protein levels in RPMI-8266 cells (Figure 5A,B).

### *PDCD4* siRNA ameliorates berberine-induced inhibition of MM cell growth

Next, we examined whether *PDCD4* knockdown could rescue the cytotoxic effect of BB treatment. RPMI-8266 and U226 cells transfected with *PDCD4* or control siRNA were treated with BB for 72 h, and cell viability was assessed by MTT assays. As shown in Figure 5C,D, *PDCD4* siRNA transfection alone did not affect cell viability. However, *PDCD4* depletion significantly ameliorated BB-induced growth inhibition, indicating that PDCD4 could be a downstream effector protein in the BB-induced cell growth inhibition pathway, and that it might be a tumor suppressor in MM cells.

## Discussion

Traditional Chinese medicine is an inexpensive treatment strategy. However, for most treatments neither the underlying therapeutic mechanisms nor the molecular targets of active compounds are well defined. Here, we describe a mechanism in MM cells by which BB inhibits cell proliferation and induces apoptosis and cell cycle arrest (Figure 3) via the regulation of miR-21 (Figure 2A).

miR-21 is over-expressed in the vast majority of cancer types analyzed so far and is thus recognized as an important oncomir [27,28]. Inhibition of miR-21 suppressed cell growth in culture and tumor growth in a xenograft mouse model [29]. Recently, Medina et al. demonstrated that miR-21 over-expression leads to a pre-B malignant lymphoid-like phenotype and that inhibiting miR-21 alone induces complete tumor regression in a few days, suggesting that miR-21 is a central oncomiR in tumor formation [30]. Interestingly, MM is also a B cell neoplasm of clonal malignant cells in the bone marrow. Another group has also suggested that miR-21 as a key oncomiR in MM [29].Our results show that BB down-regulates miR-21 levels in MM cell lines (RPMI-8266 and U226) (Figure 2A), but does not affect miR-21 levels in IL6-independent human myeloid leukemia cells (HL60, NB4, and K562 cell lines; data not shown).

Bioinformatic analysis identified two putative STAT3 binding sites upstream of the miR-21 gene (Additional file 1: Figure S4A), and STAT3 mRNA levels were significantly decreased by BB (Additional file 1: Figure S4B). STAT3 has been validated to be recruited to the miR-21 regulatory region in response to IL6 [16]. Our data indicate that IL6 levels in cell supernatants were significantly reduced by BB. Exogenous IL6 could partly ameliorate BB-mediated miR-21 down-regulation (Figure 2A). Thus, in MM, down-regulation of IL6 by BB might lead to the inhibition of miR-21 transcription through STAT3 down-regulation.

Antisense oligonucleotides targeting mRNAs have been successfully used to identify miRNA functions and for the development of therapeutic agents [31-33]. As miRNAs are small nucleic acids (19–24 nt), antisense inhibition is considered to be the best and possibly the only practical approach for specific pharmacological inhibition of their function [31]. Recently, an 8-mer nucleic acid complementary to the seed region of an miRNA, called a tiny anti-miRNA, was used to antagonize miRNA function [25,26]. In this study, high levels of AMO-mir-21-FITC were detected by confocal microscopy, mainly in the cytoplasm (Additional file 1: Figure S6A), and 95.27% of cells were FITC positive at 24 h post-transfection (Additional file 1: Figure S6B). Here, we have investigated the anti-MM effects of AMO-miR-21 and compared them with those of BB.

We also verified that *PDCD4* is a direct target of miR-21 (Additional file 1: Figure S5). Like AMO-miR-21, BB treatment down-regulated miR-21 and up-regulated *PDCD4*. Intriguingly, BB and AMO-miR-21 produced almost identical effects on cell proliferation, apoptosis, and cell cycle profile (Figure 3). Therefore, we conclude that the anti-MM activity of BB might be achieved, at least in part, through the inhibition of miR-21/PDCD4 signaling.

The identification of miRNA target genes is necessary to assess the roles of aberrantly expressed miRNAs in human cancer and to subsequently develop miRNA-based gene therapies. miR-21 is strongly up-regulated in a variety of human neoplastic disorders. It can target and, therefore, can potentially regulate a number of important tumor suppressor genes (e.g., *PDCD4*, *PTEN*, *TPM1*, *SPRY*, *RECK*, and *NFIB*), which has attracted the attention of researchers in various fields [34,35]. PDCD4, a 64 kDa protein, is an important, recently identified tumor suppressor that inhibits cell transformation, translation and invasion. PDCD4 is down-regulated in several types of human cancer [36-38], and is an independent predictor of poor prognosis in renal cell carcinoma patients [39]. Consistent with this notion, we show here that both BB and AMO-miR-21 up-regulate *PDCD4* by suppressing miR-21 (Additional file 1: Figure S5A-B, Figure 2C-D). Silencing *PDCD4* with siRNA rescued BB-induced cytotoxicity (Figure 5). Furthermore, PDCD4 modulates the expression of other genes on two levels. PDCD4 affects transcription of certain genes by inhibiting the activity of specific transcription factors, such as c-Jun [40,41], Sp1 [42] and p53 [43]. In addition, PDCD4 is thought to act as a suppressor of translation. A substantial body of evidence also suggests that PDCD4 is associated with p53 mRNA and suppresses its translation, thereby maintaining a low level of p53 in unstressed cells. This suppression is abrogated due to low levels of PDCD4 after DNA damage [44]. These observations are consistent with our predictions from the bioinformatic analysis (Figure 1).

Mechanisms of drug-insensitivity or resistance are involved in the apoptotic capacity of cancer cells [45]. Almost all cytotoxic anti-tumor drugs used clinically exert their effects by inducing apoptosis [46]. It is known that tumors escape apoptotic signals by expressing anti-apoptotic proteins, such as Bcl-2 family proteins [45,46]. Our study indicated that, similar to the effect caused by AMO-miR-21 inhibition of miR-21, BB treatment significantly promoted apoptosis, demonstrating the potential of BB and of targeting miR-21 for the treatment of MM (Figure 3). Hu et al. also show that miR-21 mediates berberine-induced apoptosis in human multiple myeloma cells [47].

## Conclusions

To the best of our knowledge, our study is the first to show that the traditional Chinese medicine, BB, modulates the expression profile of many miRNAs in MM cells by down-regulating oncomirs and/or up-regulating tumor suppressor miRNAs. Our data show that BB treatment of MM cells down-regulated miR-21, probably via inhibition of IL6/STAT3, and led to the up-regulation of PDCD4, which was likely to result in suppression of the p53 signaling pathway. Therefore, BB is a therapeutic candidate for the treatment of MM (Additional file 1: Figure S10). Our work also provides new insight into the mechanisms underlying the anti-cancer effect of a traditional Chinese herbal medicine.

## Competing interest

The authors declare that they have no competing interest.

## Authors’ contributions

JF conceived and designed the experiments. XL, JG, RZ and MF performed the experiments. JF and YL analyzed the data. XZ and JF contributed reagents, materials and analytical tools. JF, YL and XZ wrote the paper. All authors read and approved the final manuscript.

## Supplementary Material

Additional file 1: Figure S1 Flowchart of Pathway Analysis. **Figure S2.** Sequences of AMO-miR-21 oligonucleotides and seed sequences in miR-21. **Figure S3.** Heat maps illustrating unsupervised clustering of mRNAs that were differentially expressed between the treated group (T-calibrated) and the normal group (N-calibrated). **Figure S4.** BB down-regulation of STAT3 mRNA level in MM cells. **Figure S5.** miR-21 directly targets the PDCD4 3′UTR. **Figure S6.** Transfection efficiency and localization of AMO-mir-21 in RPMI-8266 cells. **Figure S7.** BB and AMO-miR-21 induction of apoptosis. **Figure S8.** BB and AMO-miR-21 induction of G2-phase cell cycle arrest. **Figure S9.** KEGG analysis of p53 signaling pathways. **Figure S10.** Proposed pathway of BB inhibition of miRNA-21 in MM cells. Click here for file

Additional file 2: Table S1 Oncomirs among down-regulated miRNAs. **Table S2.** Tumor suppressor genes among up-regulated miRNAs. Click here for file
