# Supplementary material for: Integrative analysis of differential miRNA and functional study of miR-21 by seed-targeting inhibition in multiple myeloma cells in response to berberine
Source: BMC Syst Biol. 2014 Jul 7;8:82. doi: 10.1186/1752-0509-8-82 (PMC4096730; doi:10.1186/1752-0509-8-82)
Supplement: Additional file 1: Figure S1 — Flowchart of Pathway Analysis. Figure S2. Sequences of AMO-miR-21 oligonucleotides and seed sequences in miR-21. Figure S3. Heat maps illustrating unsupervised clustering of mRNAs that were differentially expressed between the treated group (T-calibrated) and the normal group (N-calibrated). Figure S4. BB down-regulation of STAT3 mRNA level in MM cells. Figure S5. miR-21 directly targets the PDCD4 3′UTR. Figure S6. Transfection efficiency and localization of AMO-mir-21 in RPMI-8266 cells. Figure S7. BB and AMO-miR-21 induction of apoptosis. Figure S8. BB and AMO-miR-21 induction of G2-phase cell cycle arrest. Figure S9. KEGG analysis of p53 signaling pathways. Figure S10. Proposed pathway of BB inhibition of miRNA-21 in MM cells. [file 1752-0509-8-82-S1.doc]

**Supplementary Figures**

**Figure S1**

Target prediction tools

MicroT

PicTar

MiRanda

MirTarget2

TsrgetScan

Validated Target tools

Mir2Disease

miRTarBase

MiRecords

miRTarBase

Input miRNA

miRNA-Target set

**Target genes of input miRNA(s)**

Biocarta pathways

KEGG pathways

GO Terms

Enriched Biocarta

pathways

Enriched GO

pathways

Enriched KEGG

pathways

MiRAN-Target: All the interaction

MiRNA-Target:2N target

Prediction web tools

Prediction the interaction

**Figure S2**


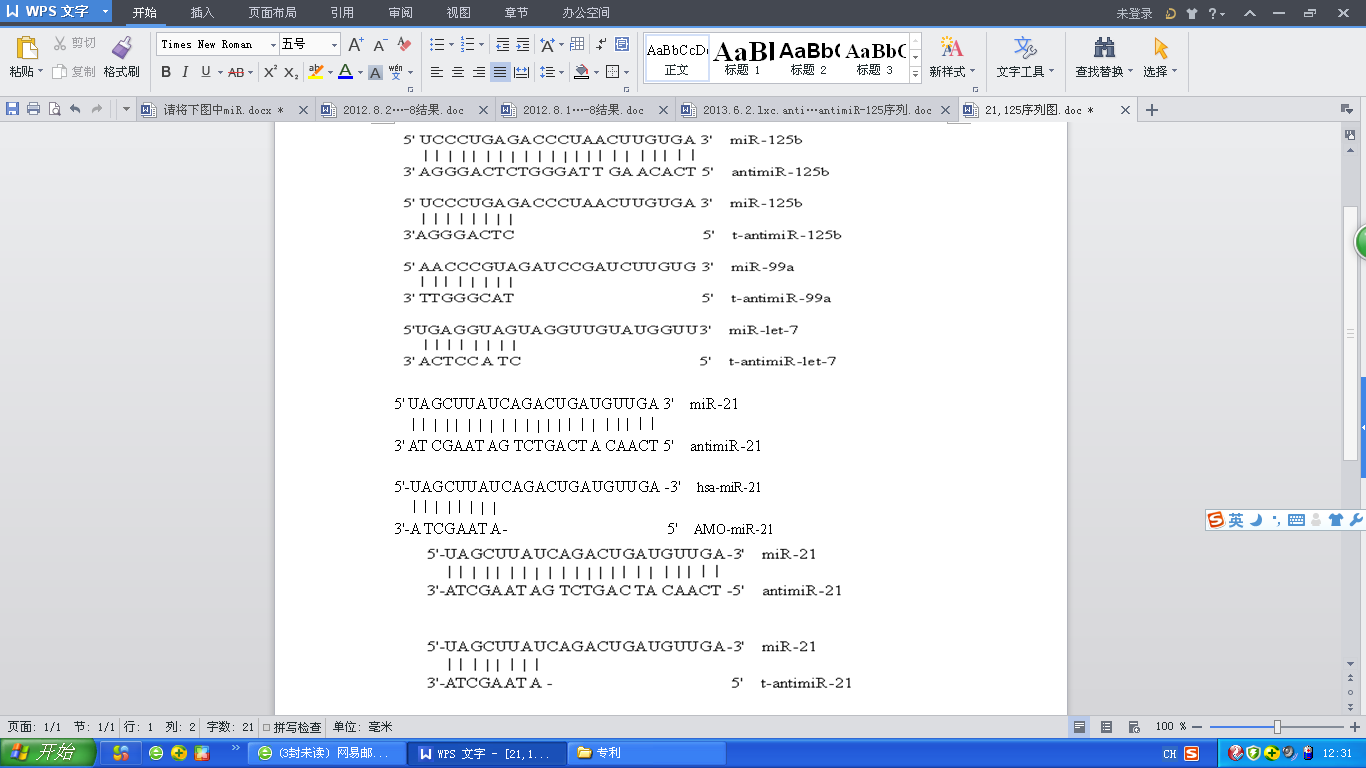


**Figure S3**

**Figure S4**


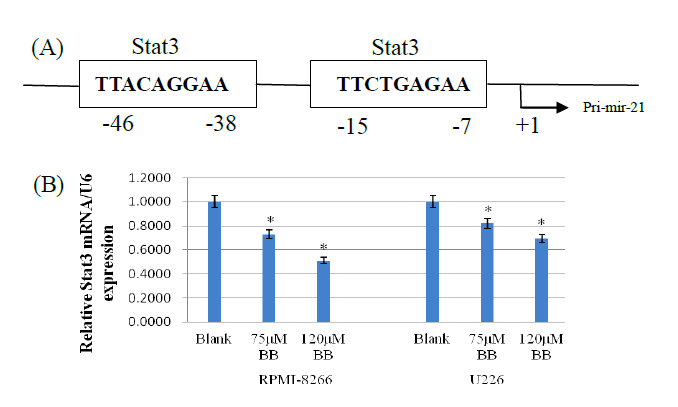


**Figure S5**


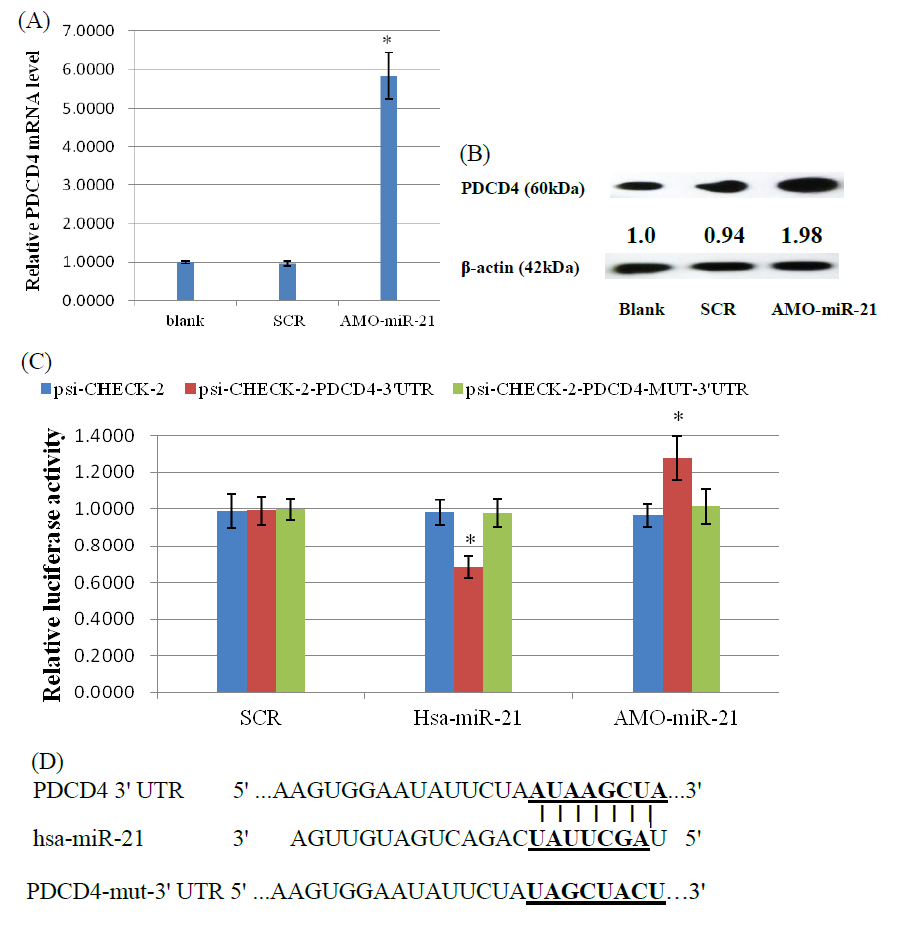


**Figure S6**


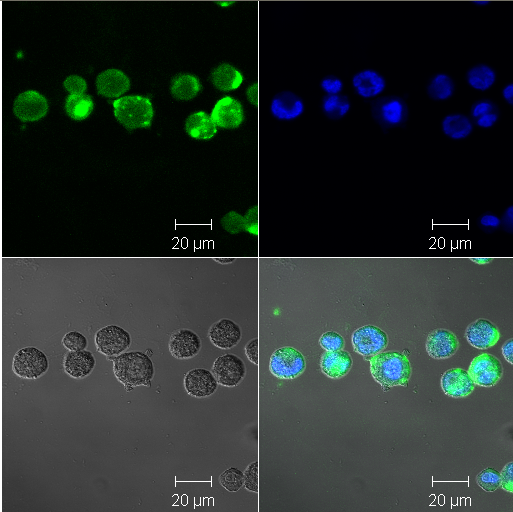

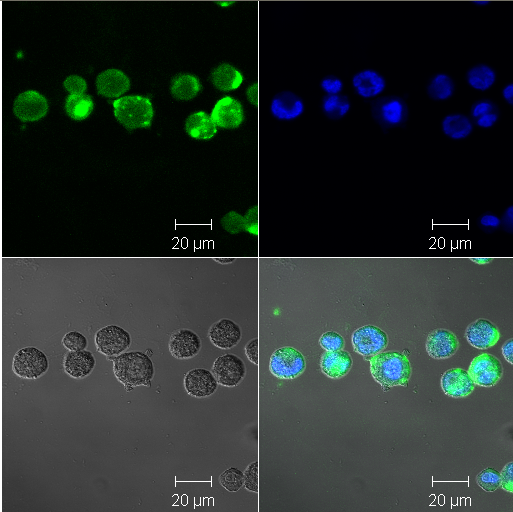

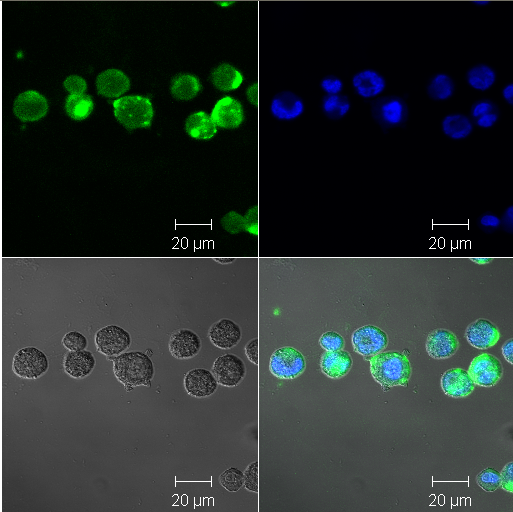


AMO-mir-21-FITC DAPI Merge

A

Blank 24h 48h


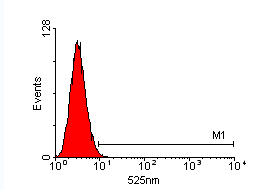


0.51%


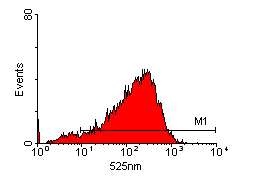


95.2%


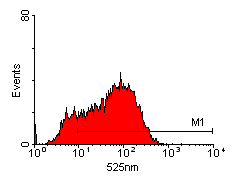


85.8%

B

**Figure S7**


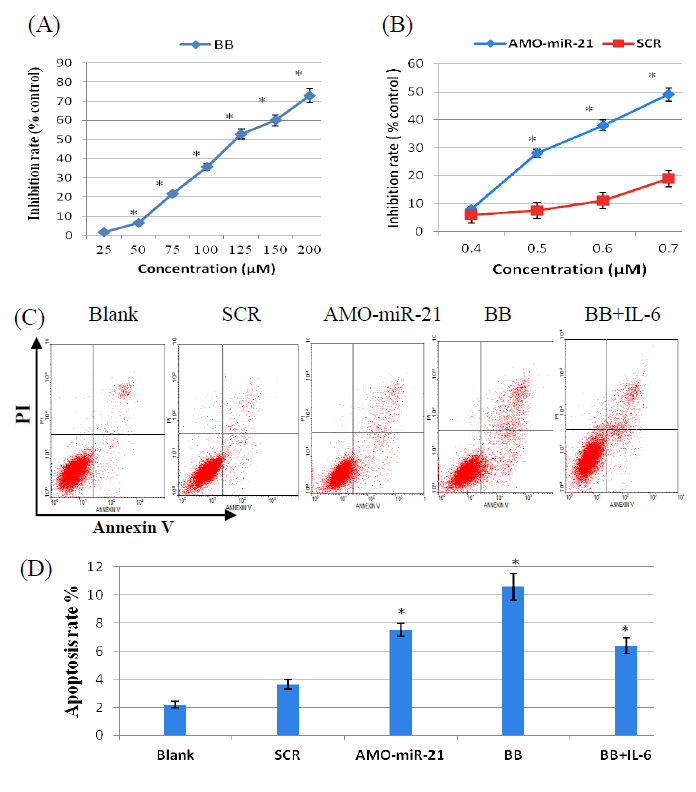


**Figure S8**


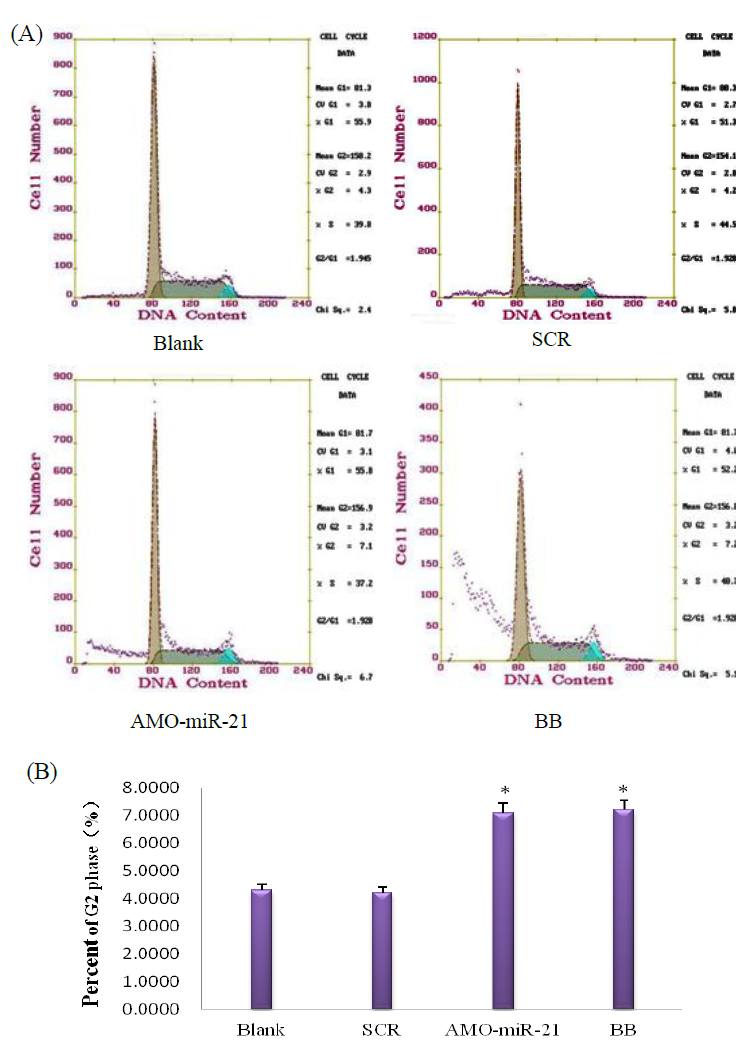


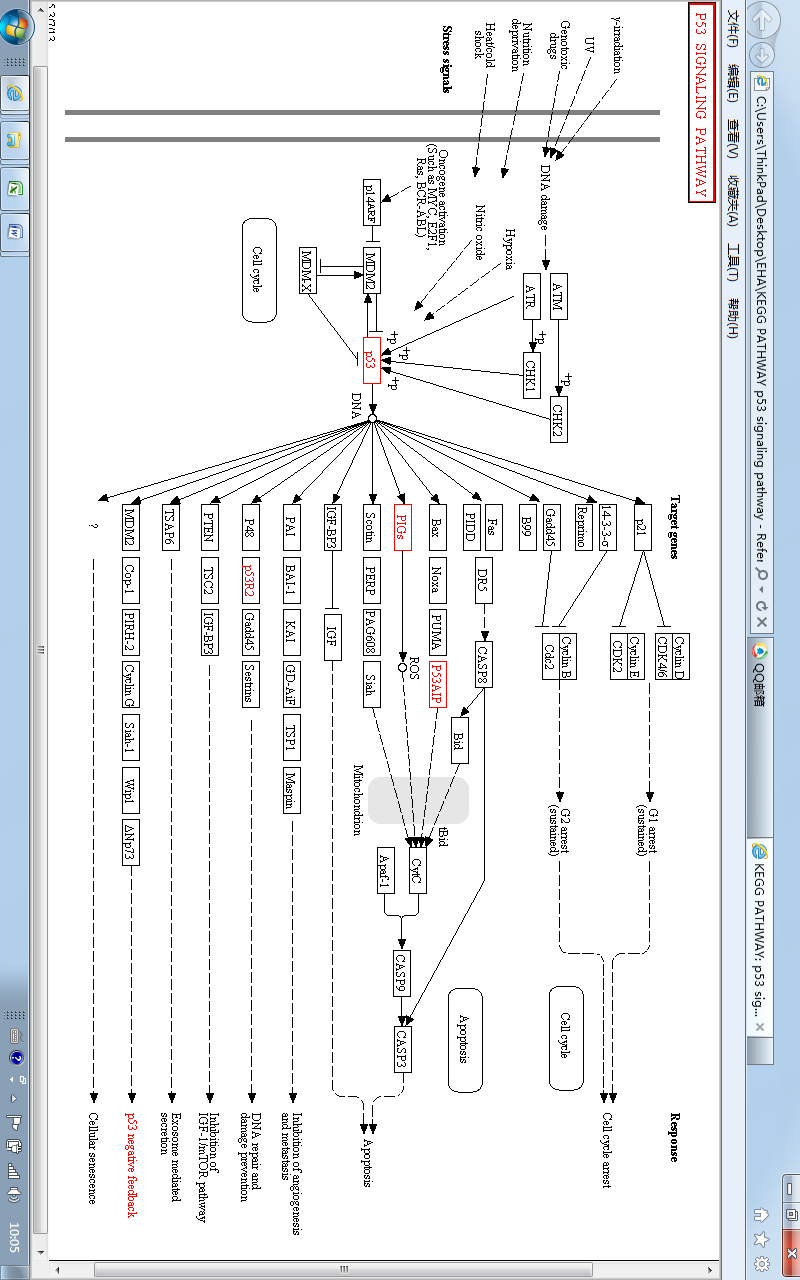


**Figure S9**

**Figure S10**


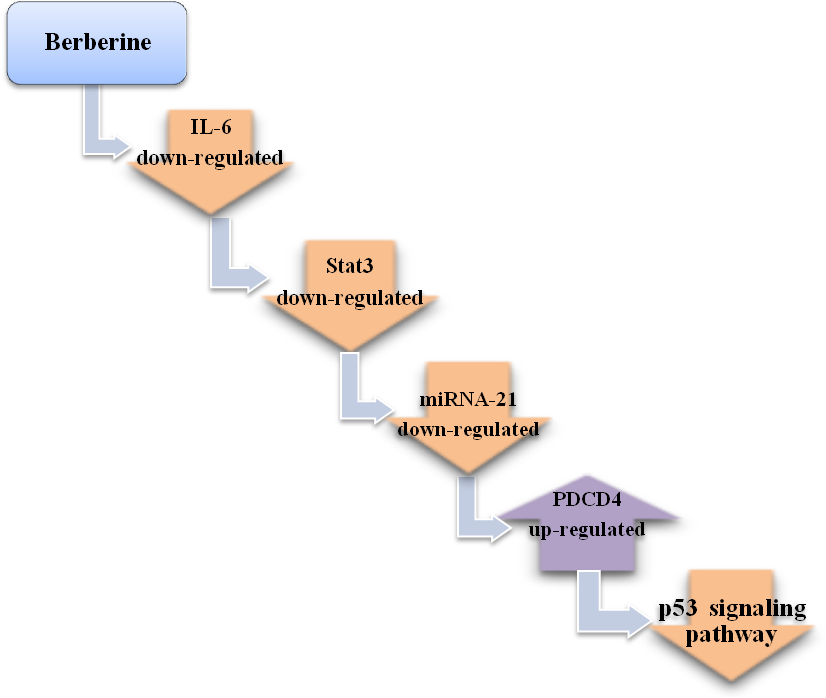


**Supplementary Legends**

**Figure S1.Flowchart of Pathway Analysis.** miRFocus software, developed by LC Science Inc, USA, intends to help miRNA researchers save valuable time in information mining once miRNA data are obtained by providing leads for in-depth analysis of miRNA-target gene pathways and the related miRNA annotations.

**Figure S2. The sequences of AMO-miR-21 oligonuleotides and seed sequences in miR-21**

**Figure S3. Heat maps illustrating unsupervised clustering of mRNAs that were differentially expressed between treated group (T-calibrated) and normal group (N-calibrated).** The green and red colors indicate relatively high and low Calibrated value-change of expression, respectively.

**Figure S4.** **BB downregulation of STAT3 mRNA level in MM cells.** (A) Schematic of miR-21 promoter fragments containing two STAT3-binding sites. DNA fragments including two putative binding sequences of miR-21 (W1: -46 to -38; W2: -15 to -7). (B) BB downregulated STAT3 mRNA level in MM cells. RPMI-8266 and U226 cells were treated with 75 μM and 120 μM BB, respectively.

**Figure S5. miR-21 directly targets the PDCD4 3'UTR.** RPMI-8266 cells transfected with scrambled or AMO-miR-21 were harvested 48 h posttransfection. Total RNA and protein were isolated, and PDCD4 mRNA (A) and PDCD4 protein (B) were determined using qPCR and Western blot, respectively. *p < 0.01 vs. control. RPMI-8266 cells were cotransfected with PDCD4-3'UTR vector or PDCD4-mut -3'UTR luciferase reporter and AMO-miR-21 (0.5 μM) or miRNA-21 mimics (100 nM). Transfection with scramble control was carried out in parallel. Renilla and firefly luciferase activities were measured by dual-luciferase reporter assay. Renilla luciferase activities was normalized to Firefly luciferase activities for each sample. (C) Overexpression of miR-21 significantly repressed Renilla luciferase activities in cells transfected with RalA-3'-UTR, and transfection of AMO-miR-21 could increase Renilla luciferase activities, but not PDCD4-mut-3'-UTR vector. Data represent the mean value of three independent experiments. *P < 0.01 vs. control. (D) Predicted binding site of miR-21 with PDCD4-3'UTR using TargetScan. Mut: contains 7-base-mutation at the miR-21 target region.

**Figure S6. Transfection efficiency and localization of AMO-mir-21 in RPMI-8266 cells.** AMO-mir-21 was modified with FITC, and transfected into RPMI-8266 cells by Lipofectamine 2000. The results indicated that the AMO-mir-21-FITC was detected to be of high level intensity and located mainly in the cytoplasm (A). flow cytometry showed that FITC positive cells were 95.27% and 85.%, respectively, at 24h and 48h post-transfection (B).

**Figure S7. BB and AMO-miR-21induction of apoptosis.** RPMI-8266 cells were treated with 75 μM BB or 0.5 μM AMO-miR-21 in the presence of Lipofectamine 2000 and serum-free RPMI-1640 for 6 h. The cells were plated in 24-well plates in medium containing 10% FCS for another 48 h. The cells were stained with FITC-conjugated annexin V and PI, followed by flow cytometry analysis. Annexin V-positive/PI-negative cells represent apoptotic cells. Both BB and AMO-miR-21 promoted apoptosis and IL-6 could overcome BB-induced apoptosis in 8266 cells. **p* < 0.01 vs. control.

**Figure S8. BB and AMO-miR-21 induction of G2-phase cell cycle arrest.** RPMI-8266 cells were treated with 75 μM BB or 0.5 μM AMO-miR-21 in the presence of Lipofectamine 2000 and serum-free RPMI-1640 for 6 h. The cells were plated in 24-well plates in medium containing 10% FCS for another 48 h. The cells were stained with PI solution and analyzed with flow cytometry. The results demonstrated that both BB and AMO-miR-21 significantly induced G2-phase arrest. **p* < 0.01 vs. blank or SCR controls.

**Figure S9. KEGG analysis of p53 signaling pathways**

**Figure S10. Proposed pathway of BB inhibition of miRNA-21 in MM cells.** In our study, the inhibitory effects of BB on MM were similar to those induced by AMO-miR-21. BB reduced miR-21 levels, possibly due to IL-6 downregulation, and led to PDCD4 upregulation, which exerts tumor suppressive effects by p53 signaling pathway in MM cells.
