# Supplementary material for: Integrative analysis of differential miRNA and functional study of miR-21 by seed-targeting inhibition in multiple myeloma cells in response to berberine
Source: BMC Syst Biol. 2014 Jul 7;8:82. doi: 10.1186/1752-0509-8-82 (PMC4096730; doi:10.1186/1752-0509-8-82)
Supplement: Additional file 2: Table S1 — Oncomirs among down-regulated miRNAs. Table S2. Tumor suppressor genes among up-regulated miRNAs. [file 1752-0509-8-82-S2.doc]

**Tables S1,2**

**Table S1. Oncomirs among down-regulated miRNAs.**

| **microRNA** | **Tumor type System Reference** |
| --- | --- |
| **miR-21** | Pre-B cell lymphoma in vivo [1] |
| **miR-103** | Gastric cancer in vitro [2] |
| **miR-19a** | Neuroblastoma in vivo [3] |
| **miR-30a-5p** | Colorectal cancer in vivo [4] |
| **miR-155** | Hepatocellular carcinoma in vivo [5] |
| **miR-30b** | Medulloblastoma in vivo [6] |
| **miR-25** | Cholangiocarcinoma in vivo [7] |
| **miR-106a** | Gastric carcinoma in vivo [8] |
| **miR-106b** | Prostate cancer in vivo [9] |

**Table S2. Tumor suppressor genes among upregulated miRNAs**

| **microRNA** | **Tumor type System Reference** |
| --- | --- |
| **miR-451** | Acute lymphoblastic leukemia in vivo [10] |
| **let-7e** | Breast tumor in vivo [11] |
| **miR-202** | Lung cancer in vivo [12] |

**REFERENCES**

1. Medina PP, Nolde M, Slack FJ. (2010) OncomiR addiction in an vivo model of microRNA-21-induced pre-B-cell lymphoma. Nature 467(7311): 86-90.

2. Li X, Zhang Y, Zhang H, Liu X, Gong T, Li M, et al. (2011) miRNA-223 promotes gastric cancer invasion and metastasis by targeting tumor suppressor EPB41L3. [Mol Cancer Re](http://www.ncbi.nlm.nih.gov/pubmed#%23)s 9(7): 824-833.

3. Lovén J, Zinin N, Wahlström T, Müller I, Brodin P, Fredlund E, et al. (2010) MYCN-regulated microRNAs repress estrogen receptor-α (ESR1) expression and neuronal differentiation in human neuroblastoma. Proc Natl Acad Sci U S A 107(4): 1553-1558.

4. Xi Y, Formentini A, Chien M, Weir DB, Russo JJ, Ju J, et al. (2006) Prognostic values of microRNAs in colorectal cancer. Biomark Insights 2: 113-121.

5. Xie Q, Chen X, Lu F, Zhang T, Hao M, Wang Y, et al. (2012) Aberrant expression of microRNA 155 may accelerate cell proliferation by targeting sex-determining region Y box 6 in hepatocellular carcinoma. Cancer 118(9): 2431-2442.

6. Lu Y, Ryan SL, Elliott DJ, Bignell GR, Futreal PA, Ellison DW, et al. (2009) Amplification and overexpression of Hsa-miR-30b, Hsa-miR-30d and KHDRBS3 at 8q24.22-q24.23 in medulloblastoma. PLOS ONE 4(7): e6159.

7. Razumilava N, Bronk SF, Smoots RL, Fingas CD, Werneburg NW, Roberts LR, et al. (2012) miR-25 targets TNF-related apoptosis inducing ligand (TRAIL) death receptor-4 and promotes apoptosis resistance in cholangiocarcinoma. Hepatology 55(2): 465-475.

8. Xiao B, Guo J, Miao Y, Jiang Z, Jiang Z, Huan R, Zhang Y, et al. (2009) Detection of miR-106a in gastric carcinoma and its clinical significance. Clin Chim Acta 400(1-2): 97-102.

9. Ambs S, Prueitt R L , Yi M, Hudson RS, Howe TM, Petrocca F, et al. (2008) Genomic profiling of microRNA and messenger RNA reveals deregulated microRNA expression in prostate cancer. Cancer Res 68(15): 6162-6170.

10. Ju X, Li D, Shi Q, Hou H, Sun N, Shen B. (2009) [Differential microRNA expression in childhood B-cell precursor acute lymphoblastic leukemia.](http://www.ncbi.nlm.nih.gov/pubmed/19206004) Pediatr Hematol Oncol 26(1): 1-10.

11. Mitra D, Das PM, Huynh FC, Jones FE. (2011) Jumonji/ARID1 B (JARID1B) protein promotes breast tumor cell cycle progression through epigenetic repression of microRNA let-7e. J Biol Chem 286(47): 40531-40535.

12. Nymark P, Guled M, Borze I, Faisal A, Lahti L, Salmenkivi K, et al. (2011) Integrative analysis of microRNA, mRNA and aCGH data reveals asbestos- and histology-related changes in lung cancer. Genes Chromosomes Cancer 50(8): 585-597.
